# Case Report: Rare Presentation of Dentin Abnormalities in Loeys-Dietz Syndrome Type I

**DOI:** 10.3389/fdmed.2021.674136

**Published:** 2021-08-17

**Authors:** Priyam Jani, Olivier Duverger, Rashmi Mishra, Pamela A. Frischmeyer-Guerrerio, Janice S. Lee

**Affiliations:** 1 Craniofacial Anomalies and Regeneration Section, National Institute of Craniofacial and Dental Research, National Institutes of Health, Bethesda, MD, United States; 2 Department of Oral Medicine, University of Washington School of Dentistry, Seattle, WA, United States; 3 Food Allergy Research Unit, National Institute of Allergy and Infectious Diseases, National Institutes of Health, Bethesda, MD, United States

**Keywords:** Loeys-Dietz syndrome, TGFBR1 mutation, TGF-beta signaling, dentin defect, dentinogenesis imperfecta, case report

## Abstract

Loeys-Dietz syndrome type 1 (LDS1) is caused by a mutation in the transforming growth factor-beta receptor 1 (*TGFBR1*) gene. We previously characterized the oral and dental anomalies in a cohort of individuals diagnosed with LDS and showed that LDS1 had a high frequency of oral manifestations, and most affected individuals had enamel defects. However, dentin anomalies were not apparent in most patients in the cohort. In this cohort, we had identified dentin anomalies in a patient with LDS1, harboring mutation *TGFBR1* c.1459C>T (p.Arg487Trp), and in this report, we present clinical and radiographic findings to confirm the dentin anomaly. The proband had gray-brown discoloration of most teeth typical for dentinogenesis imperfecta (DI). A radiographic exam revealed obliterated or very narrow pulp canals, with maxillary anterior teeth being affected more than the posterior teeth. The son of the proband, who also has the same mutation variant, had a history of DI affecting the primary teeth; however, his permanent teeth were normal in appearance at the time of exam. *TGFBR1* is expressed by odontoblasts throughout tooth development and deletion of *TGFBR1* in mouse models is known to affect dentin development. In this report, we present a rare case of abnormal dentin in two individuals with LDS1. These dental anomalies may be the first obvious manifestation of a life-threatening systemic disease and demonstrate the variable and multi-organ phenotypic effects in rare diseases.

## INTRODUCTION

Transforming growth factor-beta (TGF-β) signaling plays a crucial role during mammalian development (1). It has been implicated to be critical to cell proliferation and maturation, including the craniofacial (2-5) and dental mineralized tissues (6, 7). More recently, mutations in the TGF-β family of genes were discovered in a cohort with Marfan-like features, and the disease was classified as Loeys-Dietz syndrome (LDS) (8). LDS is caused by mutations in genes encoding various components of the TGF-β signaling pathway (9) and is currently classified into six subtypes based on the gene involved. LDS1 (MIM# 609192) is associated with transforming growth factor-β receptor type I (*TGFBR1*), TGF-β receptor type II (*TGFBR2*) mutations are classified as LDS2 (MIM# 610168), mothers against decapentaplegic homolog 3 (*SMAD3*) mutations cause LDS3 (MIM# 613795) (10), LDS4 (MIM#61481) and LDS5 (MIM#615582) are caused by mutations in TGF-β2 (*TGFB2*) and the TGF-β3 (*TGFB3*) ligands, respectively (11-14). *SMAD2* mutations were recently found to cause LDS (LDS6, no MIM# assigned) (15).

We previously reported the oral manifestations in a cohort of 40 individuals with LDS from five subtypes (LDS1-5) and reported a high frequency of abnormal palate morphology, enamel defects, bifid uvula, or submucous cleft palate, malocclusion, dental crowding, and delayed eruption of permanent teeth (16). We concluded that individuals with LDS2 followed by LDS1 had the most severely affected oro-dental region, which is also true for the systemic manifestations reported in LDS literature. Additionally, we have also shown that these dental anomalies significantly worsen the oral health-related quality of life for individuals with LDS (17).

Dentinogenesis imperfecta (DI) is a rare hereditary condition affecting the teeth, which manifests as grayish or yellow-brown discoloration of teeth (18). The condition is characterized by abnormal dentin formation leading to weak teeth which are susceptible to fracture and breakage. DI is a common manifestation in individuals with osteogenesis imperfecta Type III or IV with mutations in Collagen type 1 (*COL1A1* or *COL1A2*) genes (19, 20). Mutations in dentin sialophosphoprotein (*DSPP*) may also lead to non-syndromic forms of DI (21). While several genes have been implicated in the manifestation of dentin abnormalities in human case reports (19, 22, 23), to date, there has been no report describing dentin abnormalities associated with the TGF-β pathway in humans. However, *TGFBR1* has been shown to play a role in dentin development in mouse models (24-26). In this report, we present a rare manifestation of dentin abnormality in two individuals with a diagnosis of LDS1. Additionally, we also present a comparison between the severity of oral manifestation and systemic manifestations in LDS1, reported in previous literature and individuals in this report.

## METHODS

### Study Participant

A 53-year-old male (II-1) with a diagnosis of LDS1 who was enrolled in the Natural History and Genetics of Food Allergy and Related Conditions study (NCT02504853) was seen in the NIDCR Dental Clinic along with his wife and 14-year-old affected son (III-1). All three participants agreed to enroll and consented to the Natural History of Craniofacial Anomalies and Developmental Growth Variants study (NCT02639312).

### Genetic Test

Targeted mutation analysis for the LDS genes panel was performed for the proband and his family by the primary care physician through GeneDX (GeneDx Inc., Gaithersburg, Maryland). Subsequently, whole exome sequencing (WES) was performed through National Institute of Allery and Infectious Diseases (NIAID) Centralized Sequencing Initiative during their visit to the National Institutes of Health.

Genomic DNA was extracted from the submitted blood specimen of both individuals and subjected to massively parallel sequencing on an Illumina sequencing system. The exonic regions, flanking splice junctions, and both 5′ and 3′ untranslated regions (UTR) were sequenced with 100 bp or greater paired-end reads. Subsequently, the reads were aligned to the human genome build GRCh37/UCSC hg19, and analysis was performed using a custom-enhanced analysis tool (SEQR). The interpretation of the variants was performed according to the ACMG guidelines (27) and the nomenclature of the identified variants is consistent with the Human Genome Variation Society (HGVS) guidelines. Confirmation of potentially relevant findings was performed using capillary sequencing or other appropriate methods. Minimum coverage is 95% > x20 for targeted genomic regions.

### Oral and Dental Evaluation

The oral and dental evaluation included a detailed clinical exam, performed by the NIH Craniofacial Anomalies Team (PJ, RM, and JSL). The intra-oral exam consisted of the inspection of the oral structures and soft tissue, including the palate, uvula, and gingiva for basic periodontal assessment without periodontal probing. The dental and hard tissue evaluation included the occlusion, eruption pattern, tooth morphology, jaw relationship, and TMJ function. A standardized oro-dental evaluation form was used in all cases.

### Radiographic Evaluation

Cone beam computed tomography (CBCT) (Planmeca Promax 3D Max, 400 μm resolution; Planmeca USA Inc., IL) was performed to further assess dental phenotype including findings such as tooth impaction, dental decay, enamel or dentin defects, and alveolar bone loss. CBCT images were used to generate panoramic x-rays and individual tooth slices using Planmeca Romexis software.

### Photography

Extraoral and intraoral photographs (Canon EOS 5D Mark II camera, Canon USA Inc., VA) were obtained for each participant. For each patient, seven intraoral photos were taken: the frontal view of dentition in occlusion, the frontal view of dentition at rest (2–3 mm leeway space), the maxillary arch, the mandibular arch, the left lateral view (maxillary and mandibular teeth in occlusion), the right lateral view, and the oropharyngeal region. Facial photos included frontal and profile views.

## CASE REPORT

### History of Present Illness

**FIGURE 1 ∣:**
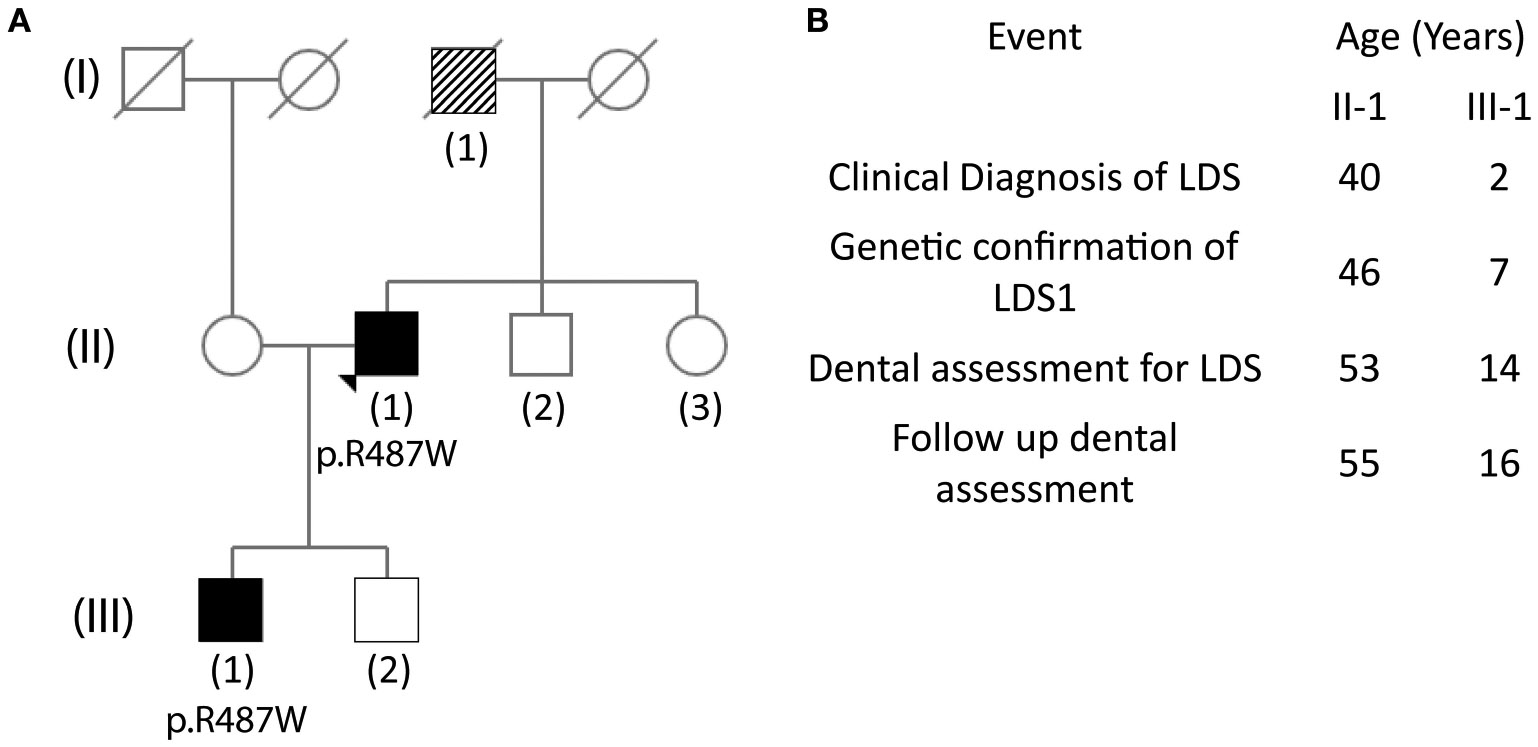
Pedigree tree showing affected family members and timeline of events. **(A)** The (II-1) father of the proband is suspected of having Loeys-Dietz syndrome (LDS) as he died from an aneurysm at age 49. The mother of the proband has early-onset dementia. The brother of the proband is healthy, and the sister has skin carcinoma. Proband has two sons, one of whom (III-1) carries the LDS mutation. **(B)** Timeline of events for the proband (II-1) and the son of the proband (III-1) since diagnosis of LDS.

The proband (II-1), a 53-year-old male, was seen at the dental clinic for evaluation of his oral health and LDS-related manifestations. He presented with his wife (unaffected, 40 years old) and son (III-1, affected, 14 years old). The timeline for proband and son are shown in Figure 1B. At age 40, the proband suffered abrupt chest and tooth pain with subsequent diagnosis of a type A aortic dissection. He underwent emergent aortic valve-sparing aortic root replacement. At age 46, his diagnosis of LDS1 was confirmed with a mutation in the *TGBR1* by candidate mutation analysis, specifically c.1459C>T, which resulted in the amino acid substitution p.R487W. At age 53, WES analysis was performed which confirmed the *TGFBR1* mutation but did not show the presence of any mutations in *DSPP*, the candidate gene for DI.

### Family History

The father of the proband (I-1) died at 49 years of age from complications related to ruptured abdominal aorta; he had a history of myocardial infarction and pulmonary embolism. Although, the father did not have a known diagnosis of LDS, it is suspected retrospectively (Figure 1A). The proband (II-1) has two sons, one of whom shares the *TGFBR1* mutation (III-1). The wife of the proband and brother are healthy, whereas, his sister has a history of skin cancer.

### Surgical History

The proband (II-1) had a history of Nissen fundoplication at age 36. He had undergone aneurysm repair at age 40 and mitral valve repair at age 51. He also had a history of inguinal hernia repair around age 45. The son of the proband (III-1) had a history of tympanostomy tube placement at age 1, inguinal hernia repair at age 9, and valve-sparing aortic root replacement at age 16. No adverse postsurgical outcomes were reported.

### Systemic Findings

The proband (II-1) had multiple systemic findings including aneurysm in aorta, aneurysm in visceral/ileac arteries, atrial fibrillation, arterial tortuosity, mitral regurgitation, joint hyperflexibility, disc degeneration, translucent and stretchy skin, inguinal hernia, hiatal hernia, eosinophilic esophagitis, GERD, pes planus, asthma, and allergic rhinitis. Several of these findings were consistent with LDS (Table 1). More recently, at age 55, the proband was also diagnosed with Waldenstrom macroglobulinemia; however, its implications to oral health were uncertain as the proband was not aware of this diagnosis at the time of exam.

The son of the proband (III-1) presented with fewer systemic manifestations which included joint hyperflexibility, scoliosis, bone fracture, food allergies, atopic dermatitis, asthma, pes planus, translucent skin, arterial tortuosity, aortic root dilation, and inguinal hernia. These findings were also consistent with LDS.

### Craniofacial/Extraoral Findings

The proband (II-1) had an oblong face with symmetrical soft and skeletal tissue. His vision was normal with the help of corrective lenses. He had significant bilateral ptosis and mild nystagmus. His midface and infraorbital projection were flat, and he was noted to have a retrognathic mandible. He had occasional temporomandibular joint (TMJ) pain and bilateral joint sounds (crepitus). He reported TMJ dislocation in the past.

The son of the proband (III-1) had an oblong face with a long lower third. He had bilateral nystagmus and mild ptosis. Both his ears were slightly lowest. He had a retrognathic mandible with normal TMJ function. He also had generalized acne on the cheeks, chin, and lips.

Overall, these patients were noted to be on the mild craniofacial spectrum compared to previous reports of craniofacial findings in LDS^1^.

### Intraoral Findings

**FIGURE 2 ∣:**
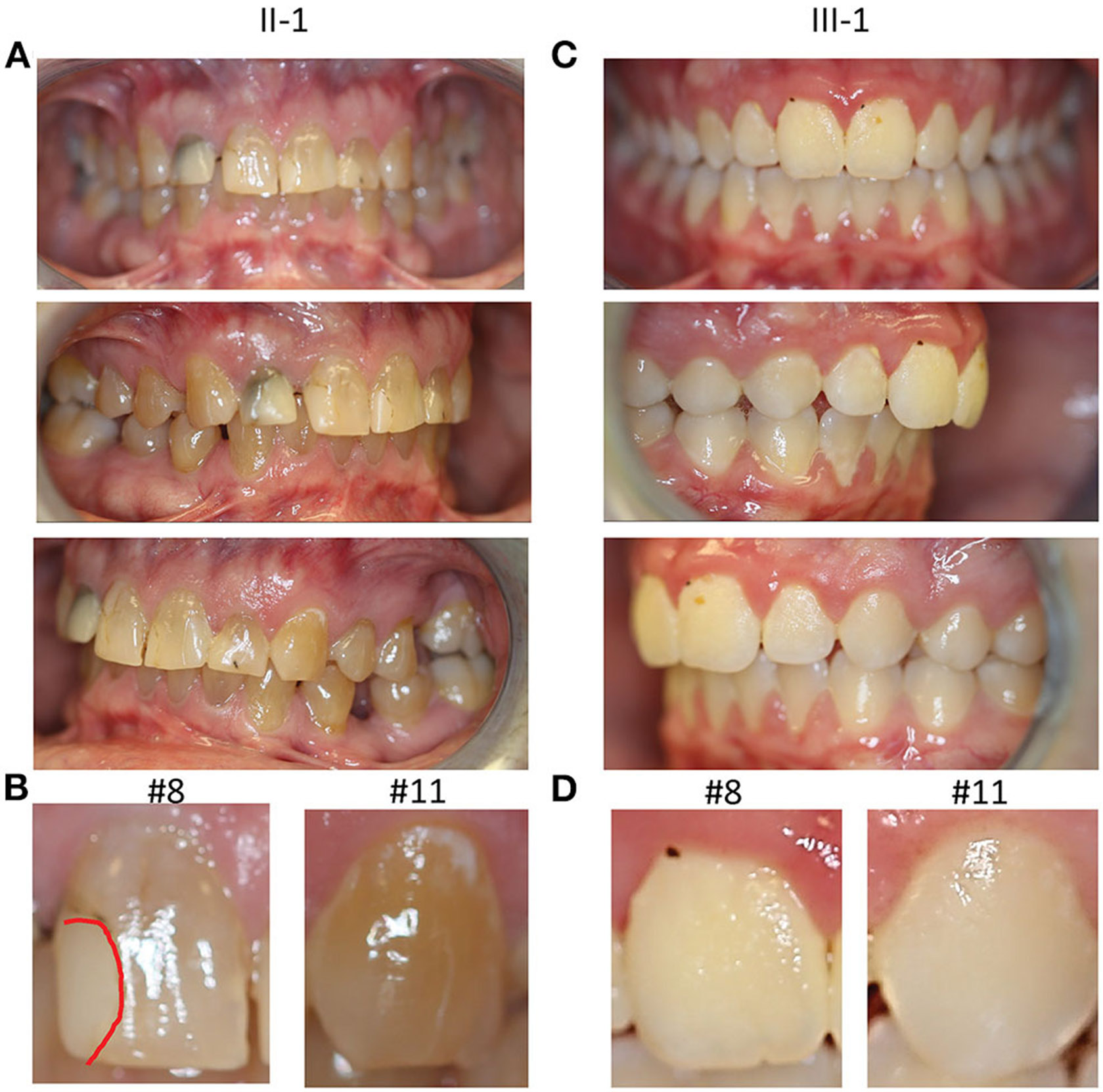
Clinical oral manifestations. **(A)** Frontal, right lateral, and left lateral view of teeth showing tooth discoloration in proband II-1. **(B)** Magnified photos of tooth #8 and 11 with a red dotted line showing demarcation of the restoration on #8. **(C)** Frontal, right lateral, and left lateral view of teeth in the son of the proband III-1 **(D)** Magnified views of tooth #8 and 11 in III-1, showing normal appearance of teeth.

The proband (II-1) had complete adult dentition with 28 teeth (wisdom teeth were extracted). He had a history of multiple cavities and had been told that his teeth are more susceptible to decay; root canal with tooth #7. His palate was high vaulted as seen in most other individuals affected by LDS. He had a Class II molar relationship with an increased overjet (7 mm) associated with mandibular retrognathia, a common feature in LDS. His teeth had grayish-brown discoloration which was unusual and prompted further investigation (Figure 2A). There were no signs of fracture or chipping of teeth, clinically, or radiographically. The discoloration affected the anterior teeth more than the posterior teeth which correlated with more restorations specifically in the anterior teeth (Figure 2B). He had mild enamel defects, grade 1 as per the enamel index (16). He did not report any bleeding of the gums and had no signs of gingivitis at the time of the exam. Mild plaque accumulation at the cervical margins of posterior teeth was noted.

The 14-year-old son of the proband (III-1) had an adult dentition with 28 teeth (Figure 2C); wisdom teeth were yet to erupt. He had a history of gray discoloration and early loss of his primary teeth. However, his permanent teeth did not have clinically discernable discoloration. He had a very mild anterior open bite with a parafunctional habit of resting his tongue in this space. He had mild crowding of teeth in the lower anterior region. He had a Class II molar relationship. He had mild generalized gingivitis and there was moderate plaque accumulation on most teeth; however, he did not report bleeding gums during routine activities like brushing his teeth. Thin occlusal enamel was noted on tooth #3, 14, 19, and 30 (first molars) with wear facets from opposing cusps which was unusual for his age. The anterior teeth did not have any apparent enamel defects, grade 0 as per the enamel index (Figure 2D). While both proband and his son had several features consistent with findings in LDS, they did not present with severe enamel defects or bifid uvula, common findings in LDS. Both individuals were seen after 2 years for a follow-up, but no changes in their oral health were noted.

### Radiographic CBCT Exam

**FIGURE 3 ∣:**
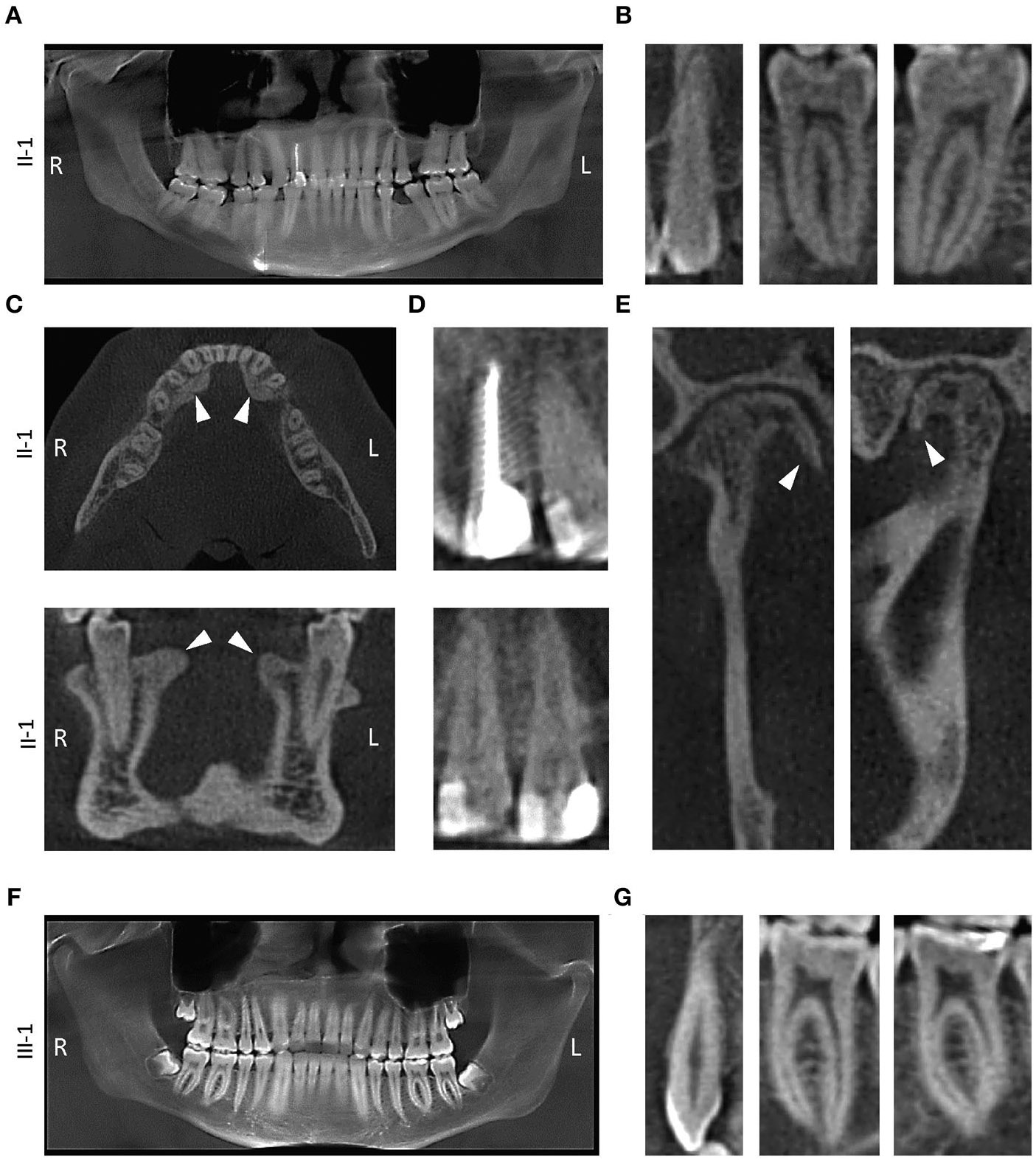
Radiographic findings. **(A)** Panoramic x-ray from proband II-1 showing thickened dentin and obstructed pulp chambers. **(B)** 2D slices from II-1 cone beam computed tomography (CBCT) exam showing tooth # 11, 21, and 30 with constricted pulp canals. **(C)** Transverse (top) and a coronal (bottom) slice of the mandible showing lingual tori (white arrowheads). **(D)** Restorations in the teeth of proband with a root canal on tooth #7 (top) and class V restorations with teeth # 8 and 9 (bottom). **(E)** Coronal (left) and a sagittal (right) slice of the right mandibular condyle showing osteophytes (white arrowhead). **(F)** Panoramic x-ray from III-1. **(G)** 2D slices showing tooth # 11, 21, and 30.

The missing teeth of proband (extractions) were confirmed in the panoramic image generated from CBCT images (Figure 3A). Bilateral tori on the medial surfaces of the mandible and small exostoses on the lateral surfaces were noted in the CBCT images (Figure 3C). Posterior teeth as well as mandibular anterior teeth showed thin pulp chambers (Figure 3B). All maxillary anterior teeth had narrow or obliterated pulp chambers indicating a phenotype mimicking DI Type II (Figure 3D). Small osteophytes involving the mandibular condyles and articular fossae were also visible explaining the TMJ abnormality experienced by the proband (Figure 3E). Mild horizontal bone loss was noted in the CBCT images in both the upper and lower arch.

The son of the proband (III-I) had developing wisdom teeth visible in the radiographic exam (Figure 3F). His teeth did have long and slender roots with somewhat constricted pulp, although, not severe enough to be considered abnormal at the time of exam (Figure 3G). His alveolar bone was within normal limits and no bone loss was seen in the CBCT scans during the exam.

### Correlation With Systemic Health

We analyzed the relationship between the systemic and oral manifestations in these two patients by linking the systemic findings (28) and oral findings (16) reported previously in LDS1 with the findings in these two patients (Table 1). While the proband (II-1) had multiple systemic conditions, several systemic findings were absent when compared to the LDS1 systemic findings reported in the literature (28). Similarly, the proband (II-1) had fewer oral manifestations compared to LDS1 affected individuals from the previous report (16), though, the DI in this individual is the first report of this dental finding in LDS. In the same fashion, the son of the proband (III-1) was on the milder spectrum of systemic and oral abnormalities, which may indicate that milder oral manifestations correlate with milder and fewer systemic conditions in individuals with LDS.

## DISCUSSION

In this case report, we show the manifestation of dentin anomalies associated with LDS1. While enamel defects have been associated with LDS, and we also showed the correlation of enamel defect severity with mutations in LDS (16), it was not identified that dentin could also be adversely affected in individuals with LDS. To our knowledge, these are the only patients with LDS1 who exhibited a phenotype recapitulating DI type II with the pathognomonic tooth discoloration in this cohort. If a larger cohort of individuals with LDS were to be thoroughly examined for oral manifestations, more reports of dentin abnormalities are possible. Thus, individuals with LDS should be screened more thoroughly, both clinically and radiographically to rule out dentin abnormalities.

One limitation of this report is that the role of *TGBR1* in causing DI phenotype could not be confirmed with just two cases. As LDS is a rare disease with arterial aneurysms as a common finding, the oral findings and symptoms are often overlooked. We have studied the oral manifestations in LDS in detail in a cohort of 40 patients, including 15 patients with LDS1, and so far, these were the only two individuals with the manifestation of dentin abnormality. The dental manifestation reported in this case study may be unique to these two individuals or as more affected individuals are studied, more details about the dental abnormalities may be revealed. Nonetheless, screening more individuals with mutations in the TGF-β signaling pathway for oral manifestations is warranted. In addition to detailed dental history and a thorough clinical exam to confirm changes in dentin, a radiographic assessment should be performed to follow the progression of the disease. Moreover, advanced genetic analysis of the two patients affected with DI in this cohort will be necessary to investigate the presence of additional mutations that may be involved in this dental manifestation which is not common in LDS but is beyond the scope of this report.

The involvement of TGF-β signaling in dentin development is supported by numerous studies in mice (7, 29, 30). Odontoblasts are the cells that form dentin and line the inner wall of the pulp cavity. TGF-β ligands and receptors are known to be expressed by odontoblasts (26, 31, 32), and stimulate the secretion of predentin and dentin. The expression of TGF-β receptor 1 in human teeth has also been reported (32). These studies suggest that TGF-β signaling does play a vital role in dentin development. While we can confirm the dentin abnormality in these patients clinically and radiographically, further, ultrastructural and histological analysis of the teeth is necessary to investigate the effects of LDS-causing mutations on dentin formation. However, extraction of teeth is not an option unless clinically indicated. The son of proband did not clinically show the discoloration of his permanent teeth despite having a history of grayish discoloration of primary teeth. However, we suspect that the DI could be progressive and as the patient ages, his teeth may eventually discolor after pulp constriction.

The two individuals in this case report do not have the many systemic manifestations as other reported cohorts with LDS1 and their craniofacial findings are also on the milder spectrum for LDS (see footnote 1). Similarly, the patients presented fewer and milder oral manifestations compared to the LDS1 cohort in the previous study (16). This suggests that the severity of oral manifestations may be related to the severity of systemic manifestations and a more thorough analysis of the correlations between oral health and systemic health is warranted. This report further emphasizes the vital role of diagnosing oral and dental abnormalities by dentists and physicians alike, as they may reflect the overall systemic health of an individual. Dental care providers could be the first to diagnose rare conditions based on the oral manifestations, following the accurate recording of history, thorough clinical exam, educating the patients about their oral conditions, and alerting the primary physicians of their findings. It is important to note that the unusual discoloration of the proband consistent with DI was likely visible well-before his aortic dissection was diagnosed and treated at age 40. As DI is relatively rare, this unusual presentation should alert clinicians and care providers that other systemic manifestations may need evaluation.

As science and medicine continue to move toward a multidisciplinary approach, dentists and oral health providers could play a critical role to advance the diagnosis, health management, and quality of life of the patient by considering the integration of oral health with systemic health and collaborating across disciplines and professions.

## Figures and Tables

**TABLE 1 ∣:** Comparison of oral manifestation with previously reported literature.

Systemic manifestation	TGFBR1 %	II-1	III-1	Oral manifestation	TGFBR1 %	II-1	III-1
Arterial tortuosity	75–100	+	+	Abnormal palate	100.0	+	+
Aortic root aneurysm	75–100	+	+	Retrognathic mandible	92.3	+	+
Pectus deformity	50–75	−	−	Gingivitis	61.5	−	+
Scoliosis	50–75	−	+	Class II malocclusion	61.5	+	−
Arachnodactyly	50–75	+	−	Enamel defects	46.1	+	−
Hernia	50–75	+	+	Submucosal cleft or bifid uvula	46.1	−	−
Osteoarthritis	25–50	−	−	TMJ abnormality	38.4	+	−
Striae	25–50	−	−	Deep bite	38.4	−	−
Dural ectasia	25–50	−	−	Dental crowding	7.6	−	+
Cervical spine malformation/instability	0–25	−	−	Delayed eruption	0	−	−
				**Dentinogenesis imperfecta**	0	+	+

−, Absent; +, Present. Systemic data for LDS1 adapted from (28); Oral data adapted from (16). Manifestation in bold are not previously reported in LDS.

## Data Availability

The original contributions presented in the study are included in the article/supplementary material, further inquiries can be directed to the corresponding author/s.

## Footnotes

1 Almpani et al., 2021, Loeys-Dietz and Shprintzen-Goldberg Syndromes: analysis of TGF-β-opathies with craniofacial manifestations using innovative characterization methods, manuscript under revision.

## Abbreviations:

LDS: Loeys-Dietz syndrome
LDS1: Loeys-Dietz syndrome type 1
TGF-β: Transforming growth factor beta
TGFBR1: TGF-β receptor type I
DI: Dentinogenesis imperfecta
CBCT: Cone beam computed tomography
